# Palladium

**Published:** 1897-01

**Authors:** Horace P. Holmes

**Affiliations:** Omaha, Neb.


### PALLADIUM.

(A paper read before the Omaha Homœopathic Medical Society.)

By Horace P. Holmes, M. D., Omaha, Neb.

I wish to call your attention to-night to a remedy that has not been discussed at any of the meetings of this society, nor has it been up for consideration at any meeting I have ever attended. We seldom see any mention of it in the homœopathic literature of the day, and yet it shows from its pathogenesis and clinical results that it should be classed with our well-known remedies, and be in almost daily use in our practice. I refer to Palladium.

Palladium is a metal found in connection with Platina, and its provings show it to be closely related to that metal as a remedy. The *American Homœopathic Pharmacopœia* says: "When the solution of crude Platinum, from which the greater part of that metal has been precipitated by sal ammoniac, is neutralized by Sodium-carbonate, and mixed with a solution of Mercuric-cyanide, Palladium-cyanide separates as a whitish, insoluble substance, which, on being washed, dried and heated to redness, yields metallic Palladium in a spongy state. The Palladium may then be welded into a mass, in the same manner as platinum."

Not only does the origin of Palladium show its close relationship to Platina, but its provings brought out so many similarities that it becomes necessary to carefully differentiate between the two. Dr. Hering suggested its use where Platina had seemed to be indicated, yet failed on account of the mental symptoms not being in accordance.

Dr. Hering made a proving of the metal in 1850 in which thirteen persons took part, and this proving was not published until twenty-eight years after, when it appeared in the November, 1878, number of the *North American Journal of Homœopathy*. We do not find it in its regular sequence in the *Encyclopædia of Pure Materia Medica*, but we do find it in the last volume of the set. Here we find six pages devoted to it, including over 150 symptoms. By far the best pathogenesis, as would be expected, is found in Hering's *Guiding Symptoms*, where it covers nearly sixteen pages. Most writers have referred to but two features in its use: the mental and ovarian symptoms.

My attention was called to this remedy through its seeming indications in cerebral anæmia, neurasthenia, and the accompanying features of those who are overworked mentally. A feature common to my own case when mentally tired, of being very irritable, and "wanting to knock people's heads off," showed its personal application. There is great mental fatigue in the evening, averse to any mental exertion, time drags slowly, exceedingly irritable. Here the remedy is to be compared with Phosphoric-acid. It is also to be compared with Pulsatilla, in that the patient's feelings are easily injured and she cries easily. With the irritability there may be very strong language used, as in Anacardium and Veratrum-alb. Palladium has the symptom: headache disappears when fixing the attention on it; this is common to Camphor and Cicuta. The opposite condition calls for Ignatia, Oxalic-acid, and, possibly, Piper-methysticum. We find the patient is so tired that he reels when coming into the room, which shows the extent of the cerebral exhaustion and the similarity to Phosphoric-acid.

The symptom—sensation as if the brain were swung from behind forward, as if the brain were shaken—I have verified on myself after taking a dose of the 200th. The characteristic headache is across the top of the head from one ear to the other. Nitric-acid has this same symptom. The headache is better from lying down and from sleep, which shows its neurasthenic character. Epiphegus has the same relief from lying down and sleep.

A queer symptom, which Hering has seen fit to incorporate under this sympton, is "no desire for beer." One instinctively looks for no desire to steal or commit arson. However, the prover probably had his beer appetite with him, as a rule, but while under the influence of the provings this desire was lacking.

There are nausea, belching of tasteless wind, and acid eructations. Here we have another similarity to Pulsatilla. There is derangement of the stomach and whitish stools, which shows liver complications.

The abdominal symptoms are not only unique in some respects, but they show the remedy likely to be indicated in bilious colic, common colic, or enteralgia, and ovarian troubles. "In intervals peculiar pain * * * in intestines, first in left and then less marked in right side; it somewhat resembles bites, as if an animal were snapping and tearing off small portions from inside." This reminds one of one of the shapes that sat at the gates of hell in *Paradise Lost*, who "seemed woman to the waist, and fair," and whose hell-hounds would—

> "When they list, into the womb
> That bred them they return, and howl and gnaw
> My bowels, their repast."

The urinary symptoms are similar to Lycopodium. There is frequent urging to urinate with scanty urine; bloody urine; urine with red, sandy deposit, staining the vessel red.

It is in the female sexual organs that this remedy has already found its chief sphere of action. All the provings and clinical experiences thus far fixes it as a right-sided remedy. The right ovary is affected. There is swelling, soreness and induration of right ovary, with shooting pains from navel to pelvis. The right ovary is sore to pressure, with bearing-down pains. The pain is worse from standing and motion, and better from rubbing and from lying down. The mental symptoms are usually present with the ovarian trouble. With the ovaritis there may be frequent urging to urinate. There is heavy weight in pelvis, and bearing down is a prominent symptom. There is soreness in abdomen after menstruation, with fear and apprehension that something horrible will happen. Yellow leucorrhœa, and also transparent, jelly-like leucorrhœa, worse before and after catamenia.

The chest symptoms are usually right-sided; there are stitches in the chest; worse from taking a deep inspiration, and better from walking in the open air. This reminds us of Bryonia. There is aching, lameness, and numbness in the limbs, again on the right side, and shows poor circulation and rheumatic indications. There is tendency for the arms to go to sleep.

Wakefulness until two o'clock in the morning is a confirmed symptom.

The pains are better from rubbing, rest, sleep, warmth, and open air; and worse from standing, motion, mental exertion, and excitement.

Cinchona has antidoted the diarrhœa, and Belladonna and Glonoine the headache. Platina is its complement.

